# Oral Semaglutide in Type 2 Diabetes: Clinical–Metabolic Outcomes and Quality of Life in Real-World Practice

**DOI:** 10.3390/jcm13164752

**Published:** 2024-08-13

**Authors:** Paola Pantanetti, Vanessa Ronconi, Marco Sguanci, Sara Morales Palomares, Stefano Mancin, Francesco Carlo Tartaglia, Giovanni Cangelosi, Fabio Petrelli

**Affiliations:** 1 Unit of Diabetology, Asur Marche–Area Vasta 4 Fermo, 63900 Fermo, FM, Italy; paola.pantanetti@sanita.marche.it (P.P.); giovanni.cangelosi@virgilio.it (G.C.); 2 Units of Diabetology and Metabolic Diseases, Ast Ancona, 60044 Fabriano, AN, Italy; vanessa.ronconi@sanita.marche.it; 3 A.O. Polyclinic San Martino Hospital, Largo R. Benzi 10, 16132 Genova, CS, Italy; sguancim@gmail.com; 4 Department of Pharmacy, Health and Nutritional Sciences (DFSSN), University of Calabria, 87036 Rende, CS, Italy; saramora17@yahoo.es; 5 IRCCS Humanitas Research Hospital, 20089 Rozzano, MI, Italy; 6 Department of Biomedical Sciences, Humanitas University, 20072 Pieve Emanuele, MI, Italy; francesco.tartaglia@st.hunimed.eu; 7 School of Pharmacy, Polo Medicina Sperimentale e Sanità Pubblica, 62032 Camerino, MC, Italy; fabio.petrelli@unicam.it

**Keywords:** obesity, oral semaglutide, type 2 diabetes, body composition, real-world evidence

## Abstract

**Background**: Glucagon-like peptide-1 receptor agonists (GLP-1 RAs) are a novel class of incretin mimetics for treating type 2 diabetes (T2D). This study evaluated the impact of semaglutide, the first oral GLP-1RA, on glycated hemoglobin (HbA1c), fasting plasma glucose (FPG), and body composition and anthropometric parameters. Additionally, the effects on cardiovascular risk factors and quality of life (QoL) in T2D patients were assessed. **Methods**: A prospective observational study with a six-month follow-up was conducted. Clinical parameters, including HbA1c, FPG, anthropometric measurements, blood pressure, cardiovascular risk factors, Diabetes Treatment Satisfaction Questionnaire (DTSQ) responses, and Short Form (36) Health Survey (SF-36) responses, were collected at baseline (T0) and at six months (T1). **Results**: Sixty-one subjects were enrolled, with there being an average T2D duration of 4.67 ± 3.93 years. Significant decreases were observed in HbA1c (µ = −1.24; SD = 1.33; *p* < 0.05), FPG (µ = −31.01 mg/dL; SD = 41.71; *p* < 0.05), body composition and anthropometric parameters (*p* < 0.05), and cardiovascular risk factors (*p* < 0.05), with an increase in DTSQ scores (*p* < 0.05). **Conclusions**: The administration of 14 mg/day oral semaglutide improved several clinical parameters after six months of treatment. These findings suggest semaglutide is effective in improving glycemic control, weight management, and some cardiovascular risk factors in T2D patients.

## 1. Introduction

Type 2 diabetes mellitus (T2D) represents a major global health challenge, significantly impacting morbidity and mortality rates. According to the World Health Organization (WHO), the number of individuals affected by diabetes has quadrupled over the past thirty years [1]. The International Diabetes Federation estimates that the prevalence of diabetes, which was 10.5% in 2021, will increase to 11.3% by 2030 and 12.2% by 2040 [2]. T2D is closely associated with factors such as body mass index (BMI) and waist circumference (WC), with both being indicators of central adiposity. Elevated BMI and increased WC are linked to a higher risk of developing T2D, and reducing these parameters is crucial for disease management and the prevention of associated complications [3].

Several pharmacological approaches are available for treating T2D. Among them, glucagon-like peptide-1 receptor agonists (GL-1RAs) are considered to have a very high efficacy for not only glucose lowering but also for addressing other cardiometabolic risk factors [4,5]. These agents are known as incretin mimetics. They act by increasing the level of the GLP-1 hormone in a physiological and pharmacological way, thereby reducing the risk of hypoglycemia complications [6]. Current national and international guidelines for T2D management recommend the use of GLP-1 RAs as a first- or second-line treatment (after metformin) due to their beneficial effects on body weight (BW) and cardiorenal risk [7,8]. BW reduction, blood pressure (BP) and lipid profile (LP) improvement, and anti-inflammatory and anti-atherosclerotic properties [9,10] may explain the ability of GLP-1RAs to reduce cardiovascular and renal endpoints beyond glycemic control (GC) [11,12]. Recently, it was found that the quality of life (QoL) of clients seems to benefit from the use of subcutaneous semaglutide formulas in real-world clinical practice, even one year on from beginning the treatment [13]. Semaglutide is the first oral GLP-1RA: the co-formulation with the absorption enhancer sodium N-[8-(2-hydroxybenzoyl) amino caprylate] (SNAC) allows for a localized increase in pH, protecting semaglutide against proteolytic degradation and increasing its bioavailability [14]. Once-daily oral semaglutide was developed to facilitate the initiation of and adherence to treatment with a GLP-1 receptor agonist. Indeed, there is evidence that, especially in injection-naive patients, adherence to oral therapy is greater than that for injectables [15]. Oral semaglutide has undergone extensive phase 3 clinical testing: the PIONEER program (Peptide Innovation for Early diabetes treatment) comprised trials comparing once-daily oral semaglutide with a placebo or active comparators in different populations [16,17,18,19,20,21,22,23,24], also including a Cardiovascular Outcome Trial (CVOT) [25]. The PIONEER program showed that oral semaglutide was non-inferior to the placebo in terms of cardiovascular safety, and a significant improvement in GC, BW, BMI, and WC in the studied subjects was noted [26]. While several studies [27,28,29,30] have provided valuable insights, it is crucial to expand real-world evidence to validate clinical trial results in diverse patient populations and everyday clinical settings. Additionally, a deeper understanding of the impact of oral semaglutide on GC, weight management, cardiovascular risk factors, and QoL is essential for optimizing treatment strategies for T2D.

### Study Objective

This study evaluated the impact of oral semaglutide on glycated hemoglobin (HbA1c, %), fasting plasma glucose (FPG, mg/dL), and body composition and anthropometric parameters including BW (kg), BMI (kg/m^2^), body water (kg), fat mass (%), and muscle mass (%). Secondarily, the effects on cardiovascular risk factors such as systolic blood pressure (SBP, mmHg), diastolic blood pressure (DBP, mmHg), total cholesterol (TC, mg/dL), low-density lipoprotein cholesterol (LDL, mg/dL), high-density lipoprotein cholesterol (HDL, mg/dL), and triglycerides (TG, mg/dL) and QoL, measured by the Short Form (36) Health Survey (SF-36) and the Diabetes Treatment Satisfaction Questionnaire (DTSQ), in T2D patients were assessed.

## 2. Materials and Methods

### 2.1. Study Design

This was a double-center single-arm observational prospective clinical study conducted in the Diabetes and Nutrition Clinic at the tertiary hospitals in Ast Fermo, Italy, and Ast Ancona, Italy.

### 2.2. Ethical Considerations

All procedures followed were in accordance with the responsible committee’s ethical standards on human experimentation (institutional and national) and the Declaration of Helsinki. The study protocol was approved by the CERM Ethics Committee, protocol n. 2022/123, on 30 June 2022. An informed consent form was signed by all participants.

### 2.3. Sample and Criteria

All patients initiating oral semaglutide treatment in the quarter between July 2022 and October 2022 were included.

The inclusion criteria were as follows: T2D diagnosis, men or women, age ≥ 18 years, need for therapy intensification based on the physician’s judgment, and signed informed consent.

The exclusion criteria were as follows: intolerance to or contraindications for oral semaglutide, previous GLP-1 RA or SGLT2i therapy, concomitant or suspected malignant diseases, pregnancy/breastfeeding, recent (within 3 months of enrolment visit) acute illnesses (except viral illnesses), renal impairment (eGFR < 60 mL/min), severe liver failure, congestive heart failure (NYHA IV classes), proliferative diabetic retinopathy, presence of cholelithiasis, chronic pancreatitis or ongoing acute pancreatitis, and a ketogenic diet.

### 2.4. Study Procedures

According to standard care, oral semaglutide was prescribed at starting doses of 3 mg/day during the first month of therapy, 7 mg/day during the second month, and 14 mg/day for the following months up to 6 months. According to the clinical practice of the diabetes clinic, patients received a nutritional intervention based on the following nutrient intake percentages: carbohydrates 45–60%, proteins 10–20%, fats 25–30%, and a recommended caloric intake of 25–30 Kcal/kg (ideal weight).

At baseline (T0), the following patient information was collected: age, gender, T2D duration, HbA1c (%), FPG (mg/dL), BW (kg), BMI (kg/m^2^), body water (kg), fat mass (%), muscle mass (%), SBP (mm/hg), DBP (mm/hg), TC (mg/dL), LDL (mg/dL), HDL (mg/dL), and TG (mg/dL). Furthermore, a routine Bioelectrical Impedance Analysis (BIA) was performed (DC-430MA™, TANITA Europe BV, Amsterdam, The Netherlands) to measure body composition, body water (kg), fat mass (%), and muscle mass (%).

Finally, the DTSQ and the SF-36 were used to evaluate QoL and treatment during care. Data on clinical parameters, BIA, and the questionnaires were also collected after 6 months (T1) following the initiation of oral semaglutide treatment (T0). The DTSQ is specifically designed to measure satisfaction with diabetes treatment regimens [31]. It is composed of eight items, six of which are summed in a single score ranging from 0 (very dissatisfied) to 36 (very satisfied). The remaining two items are treated individually and explore the perceived frequency of hyperglycemic and hypoglycemic episodes, with higher scores indicating a higher frequency. The Italian version was adopted for this study [32]. The SF-36 is one of the most widely used measures of health-related quality of life (HRQOL) and consists of 36 items covering eight dimensions: physical functioning, role limitations caused by physical health problems, bodily pain, general health perception, vitality, social functioning, role limitations caused by emotional health problems, and mental health [33]. The SF-36 has been used in large-population studies and in many different clinical contexts, facilitating relevant psychometric outcomes [34,35]. It has been translated into, and validated in, several languages, including Italian [36].

### 2.5. Statistical Analysis

To perform the statistical analysis, the dataset was first uploaded and loaded into a pandas Data Frame. The missing values (NaNs) were filled with the mean of their respective columns to ensure that the statistical tests could be performed without any data gaps. The dataset included various health and clinical parameters measured at baseline and after 6 months of treatment with oral semaglutide (14 mg/day). To determine which statistical test to use, we conducted normality tests for each variable using the Shapiro–Wilk test and the Kolmogorov–Smirnov test. If the data were normally distributed (as indicated by these tests), a paired-samples *t*-test was employed to compare the baseline and post-treatment values of the clinical parameters. If the data were non-normally distributed, the Wilcoxon signed-rank test was used. The paired-samples *t*-test assesses whether the mean difference between these pairs is significantly different from zero, while the Wilcoxon signed-rank test assesses whether the median difference between pairs is significantly different from zero. For the analysis, Python SciPy Stats was used, and for each pair of measurements, the following statistical measures were calculated: mean difference, the average difference between post-treatment and baseline values; standard deviation (SD), the dispersion of the differences around the mean difference; Standard Error of the Mean (SE Mean), the SD of the sample mean difference, which provides an estimate of the precision of the mean difference; 95% Confidence Interval (CI), the range within which the true mean difference lies with 95% confidence; Test Statistic, the Test Statistic calculated for the paired-samples *t*-test or Wilcoxon signed-rank test; *p*-value, the probability of observing the data assuming the null hypothesis is true. A *p*-value less than 0.05 indicated a statistically significant difference.

## 3. Results

### 3.1. Sample

**Figure 1:**
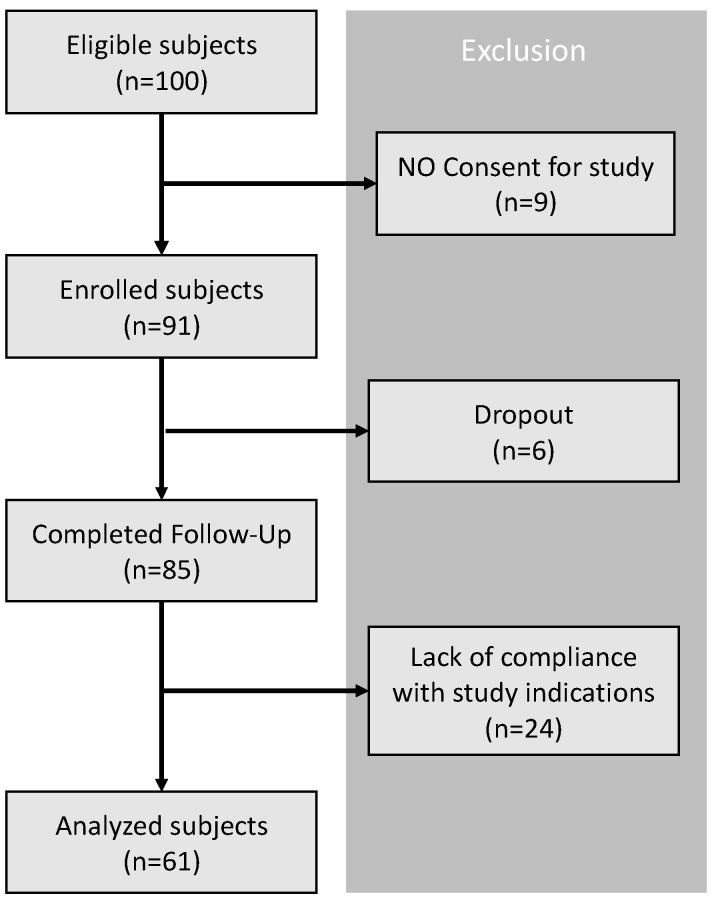
Chronological study flow chart.

Out of the 100 eligible patients who met the inclusion criteria in routine real-life care, 91 agreed to participate in the study. Among these, 6 patients were excluded because they dropped out, leaving 85 patients who completed the required follow-up. Of these, 24 were excluded due to a lack of compliance with study protocols, resulting in a final sample of 61 patients (Figure 1).

**Figure 2:**
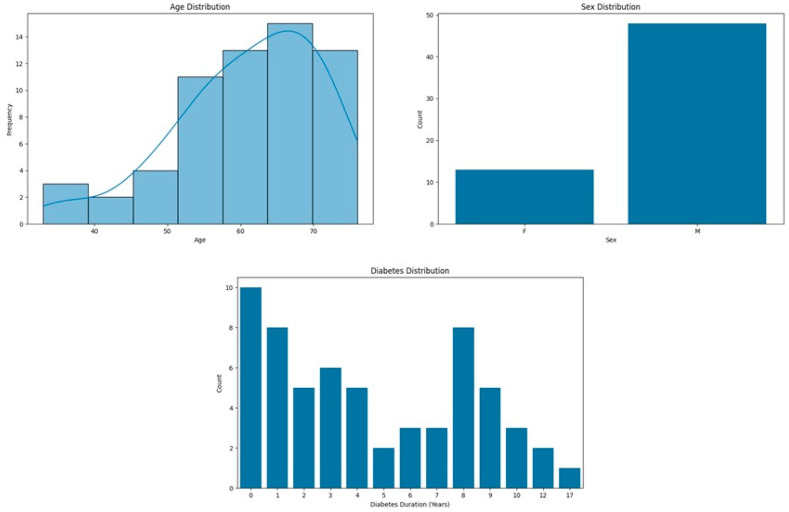
General characteristics of the sample.

In this study, a once-daily oral dose of 14 mg semaglutide was administered. A sample of 61 adult patients was recruited, 48 of whom were male (61% of the eligible cohort) with a mean age of 61 years SD ± 9.90 (range 33–76 years) and an average T2D duration of 4.67 years SD ± 3.93 (range 0–17 years). A general description of the characteristics of the sample is detailed in Figure 2.

### 3.2. Baseline Characteristics of Study Participants

At baseline (t0), the participants presented with various parameters; we analyzed them and divided them into three main categories: glycemic and anthropometric parameters and body composition, cardiovascular risk factors, and QoL and treatment satisfaction.

#### 3.2.1. Glycemic and Anthropometric Parameters and Body Composition

The participants had a mean HbA1c of 7.92% SD = 1.33. The mean FPG was 154.33 mg/dL SD = 41.71. The mean BW was 89.19 kg SD = 5.84, while the mean BMI was 30.81 SD = 1.96, with 56.67% of patients being obese (BMI ≥ 30). The mean body water content was 44.68 kg SD = 6.14, the mean fat mass was 30.81% SD = 8.01, and the mean muscle mass was 59.99% SD = 8.74.

#### 3.2.2. Cardiovascular Risk Factors

The mean SBP was 138.85 mmHg SD = 16.53, and the mean DBP was 82.14 mmHg SD = 12.04. The mean value for TC levels was 182.05 mg/dL SD = 46.26, while the mean LDL cholesterol was 113.5 mg/dL SD = 34.51. The mean HDL cholesterol was 45.07 mg/dL SD = 9.11, and the mean value for TG levels was 192.68 mg/dL SD = 115.11.

#### 3.2.3. QoL and Treatment Satisfaction

The mean QoL score, measured by the SF-36, was 100.02 SD = 14.79, while the mean DTSQ score was 26.7 SD = 8.75.

### 3.3. Impact of Semaglutide on Glycemic and Anthropometric Parameters and Body Composition

After 6 months of taking oral semaglutide at a dosage of 14 mg/day, there was a significant mean decrease in HbA1c levels from baseline, with a reduction of −1.24% SD = 1.33; *p* < 0.05. BW showed a mean weight loss variation of −3.09 kg SD = 5.84; *p* < 0.05. There were relative improvements in BMI, with a mean reduction of −1.19 SD = 1.97; *p* < 0.05. Clinical parameters also indicated a statistically significant decrease in FPG, with a mean variation at 6 months of −31.01 mg/dL SD = 41.71; *p* < 0.05. There was a significant change in fat mass percentage, with a mean change of −0.07% SD = 8.01; *p* < 0.05. The results are summarized in Table 1.

### 3.4. Impact of Semaglutide on Cardiovascular Risk Factors

Regarding the regression between the administration of semaglutide at a dosage of 14 mg/day and the main cardiovascular risk factors, the study showed a mean decrease in SBP of −12.74 mmHg SD = 16.53; *p* < 0.05 and a mean decrease in DBP of −6.39 mmHg SD = 12.04; *p* < 0.05. Also, TC levels decreased by a mean of −22.19 mg/dL SD = 46.26; *p* < 0.05. LDL levels showed a mean reduction of −18.00 mg/dL SD = 34.51; *p* < 0.05. On the other hand, HDL levels showed no significant variation. The results are summarized in Table 2.

### 3.5. Impact of Semaglutide on QoL and Treatment Satisfaction

The QoL and SF-36 scores were assessed using the Wilcoxon signed-rank test. The QoL measures showed no significant change in terms of SF-36 score, with a mean change of 1.16 SD = 14.79; *p* = 0.17. In contrast, the DTSQ demonstrated a significant improvement, with a mean change of 4.31 SD = 8.75; *p* < 0.05 (Table 3).

## 4. Discussion

This study evaluated the impact of oral semaglutide on HbA1c, FPG, body composition, and anthropometric parameters. Additionally, the effects on cardiovascular risk factors and QoL in T2D patients were assessed. Our findings demonstrated significant improvements in several metabolic and clinical parameters over a six-month period of treatment with oral semaglutide 14 mg/day.

Regarding the change in HbA1c levels from baseline to 6 months, we observed a mean decrease of −1.24% (*p* < 0.05). This reduction in HbA1c is clinically significant, as it indicates improved long-term glycemic control, which is essential for reducing the risk of diabetes-related complications [37]. Previous studies have similarly shown that lowering HbA1c levels is associated with a decreased risk of microvascular and macrovascular complications in patients with T2D [38,39]. The observed decrease in FPG of −31.01 mg/dL (*p* < 0.05) further supports the efficacy of semaglutide in improving GC. Consistent FPG reduction is critical for the daily management of diabetes and prevents acute hyperglycemic episodes [40].

This study also documented significant weight loss among participants, with a mean decrease of −3.09 kg (*p* < 0.05). This weight loss was reflected in a reduction in BMI of −1.19 (*p* < 0.05). Weight management is a crucial component in the treatment of T2D, as excess weight and obesity are major risk factors for the development and progression of the disease [41]. The reduction in BMI and BW aligns with findings from other studies that have demonstrated the efficacy of GLP-1 receptor agonists in promoting weight loss in T2D patients and those at risk [42,43]. Body fat percentage reduction, although not statistically significant in our study, is an important indicator of improved body composition and metabolic health. It is worth noting that the observed decrease in body fat percentage was minimal, as was that for other parameters such as muscle mass and water content. Loss of muscle mass during weight reduction is a common occurrence and can be attributed to overall weight loss. Muscle mass is closely related to water content, as muscle tissue contains a high percentage of water [44]. Thus, a decrease in muscle mass can lead to a concomitant reduction in total body water percentage [45]. In our study, we observed a mean decrease in muscle mass of 2.07% (SD = 8.74, SE Mean = 1.12), with a *p*-value of 0.06, indicating a trend towards significance. Additionally, body water decreased by 1.66 kg (SD = 6.14, SE Mean = 0.79, WSRT Test Statistic = 523, *p*-value < 0.05). This suggests that the reduction in muscle mass may have contributed to the observed decrease in body water. This relationship highlights the importance of monitoring muscle mass and hydration status in patients undergoing weight loss therapy to ensure that the reductions are primarily related to fat mass rather than lean tissue.

Cardiovascular risk management is a crucial component in the treatment of T2D due to the high prevalence of cardiovascular disease (CVD) among diabetic patients [46]. Our study demonstrated significant reductions in both SBP and DBP. This is particularly relevant given the established link between hypertension and increased cardiovascular events in T2D patients. Hypertension is a major modifiable risk factor for CVD, and controlling BP can significantly decrease the incidence of heart disease and stroke [47].

The BP-lowering effects of GLP-1 receptor agonists such as semaglutide have been corroborated by multiple studies. For instance, the SUSTAIN 6 trial reported significant reductions in SBP and DBP in patients treated with semaglutide, highlighting its potential for cardiovascular protection beyond GC. This effect is thought to be mediated through several mechanisms, including weight loss, improved endothelial function, and reductions in arterial stiffness [48]. The PIONEER 6 trial also confirmed these findings, demonstrating significant improvements in cardiovascular outcomes with oral semaglutide [49].

In addition to BP, our study observed notable reductions in lipide profile (LP), specifically in TC and LDL levels. Elevated LDL levels are a well-known contributor to atherosclerosis and subsequent cardiovascular events. Reducing LDL levels is a primary target in cardiovascular risk management [50]. The lipid-lowering effects observed with semaglutide in our study are consistent with findings from other research, such as the meta-analysis in [51], which confirmed the efficacy of GLP-1 receptor agonists in reducing LDL cholesterol levels. These improvements in LP contribute to the overall cardiovascular risk reduction observed with GLP-1 receptor agonist therapy.

Overall, the significant reductions in BP and cholesterol levels observed in our study underscore the comprehensive cardiovascular benefits of semaglutide in managing T2D. These benefits are directly correlated with reductions in inflammation and atherosclerotic processes, as demonstrated by previous studies [52]. Moreover, the anti-inflammatory and anti-atherosclerotic properties of GLP-1 receptor agonists add another layer of cardiovascular protection. Inflammatory markers, which are often elevated in T2D patients, have been shown to decrease with GLP-1 therapy, further reducing cardiovascular risk [53].

Regarding QoL, it is an essential consideration in the management of chronic diseases like T2D, and in our study, QoL measures presented mixed results. The SF-36 score, which measures general health perceptions, showed no significant change. This lack of change in general health perception might be attributed to the short duration of the study or the multifaceted nature of QoL, which is influenced by various factors beyond GC and weight management. Conversely, the DTSQ revealed a significant improvement in patient satisfaction with diabetes treatment. This improvement is likely due to the effect on GC and the weight loss achieved with semaglutide therapy, which may improve patients’ perception of their treatment regimen, in addition to facilitating a preference for oral administration by reducing “more invasive” administrations such as subcutaneous insulin therapy. Furthermore, improved treatment satisfaction is a critical outcome, as it can lead to better adherence to therapy, essential for long-term diabetes management [54]. Previous studies have also demonstrated that GLP-1 receptor agonists, including semaglutide, improve patient satisfaction and perceived treatment efficacy. For example, the PIONEER 3 trial found that patients treated with oral semaglutide reported higher satisfaction compared to those on other antidiabetic medications [18]. Enhanced treatment satisfaction can positively impact patients’ psychological well-being and motivation to maintain their treatment regimen, leading to sustained GC and improved health outcomes [55,56].

An additional consideration lies in the prevalence of male subjects in our study. Numerous studies [57,58] have highlighted significant differences between sexes in the prevalence of T2D, progression of T2D, and response to T2D treatment. Men tend to develop T2D at a younger age compared to women and may have a greater predisposition to cardiovascular complications associated with diabetes. Moreover, hormonal differences and behavioral risk factors, such as obesity rates and physical activity, can influence susceptibility to T2D between genders. In our sample, the high percentage of male patients may reflect these epidemiological trends. It is possible that men were more frequently diagnosed with T2D at an older age and had a longer duration of the disease compared to women. However, it is important to consider that gender differences in response to semaglutide treatment were not explored in this study.

This study highlights the multiple benefits of incorporating oral semaglutide into the management of T2D. Although improvements in overall quality of life measures were not significant, the significant improvement in satisfaction with diabetes treatment indicates a positive impact on patients’ treatment experiences.

Our results are consistent with the scientific literature on the benefits of GLP-1 receptor agonists, reinforcing the value of semaglutide in comprehensive diabetes care by demonstrating not only improvements in glycemic control and weight management but also reductions in CVD risk factors and overall mortality among patients with T2D [59,60]. The alignment between our results and those in the current literature underscores the strong profile of oral semaglutide as a key therapeutic agent in modern diabetes management. Future research should continue to explore the long-term benefits of semaglutide use and potential areas for optimization in treatment protocols involving semaglutide.

### Limitations

This study has several limitations. Firstly, the sample size was relatively small, consisting of only 61 adult patients, which may affect the generalizability of the results. A larger and more diverse cohort is needed to validate these findings. Secondly, the study duration was only six months, meaning it was insufficient for observing the long-term effects and sustainability of oral semaglutide benefits. Longer follow-up periods are necessary to assess enduring impacts on GC, weight management, cardiovascular risk factors, and QoL. Thirdly, the absence of a placebo control group limits the ability to attribute the observed changes solely to the intervention. Including a control group would strengthen causal inferences regarding the effects of semaglutide. Additionally, reliance on self-reported measures for QoL and treatment satisfaction may introduce response bias. The use of objective measures alongside subjective assessments could provide a more comprehensive evaluation of the therapy’s impact. Moreover, potential confounding variables such as changes in diet, physical activity, and concomitant medications were not fully controlled for. These factors could have influenced the outcomes.

Lastly, body composition changes assessed through BIA have limitations in accuracy compared to more advanced imaging techniques. Future studies using precise methods like dual-energy X-ray absorptiometry (DEXA) would provide clearer insights.

## 5. Conclusions

This study demonstrated that a six-month treatment with oral semaglutide at a daily dose of 14 mg significantly improves GC, weight management, and cardiovascular risk factors in patients with T2D. Hb1Ac and FGP levels showed substantial reductions, and notable weight loss and BMI reduction results were observed, aligning with the known benefits of GLP-1 receptor agonists. Cardiovascular improvements included significant decreases in BP, TC, and LDL levels. QoL results were mixed, with increased patient satisfaction despite no significant change in overall health perception. Despite limitations such as a small sample size and short study duration, the findings support the efficacy of oral semaglutide in managing T2D. Future research with larger populations and longer follow-up times is needed to confirm these results and explore long-term effects. Overall, the use of oral semaglutide at a daily dose of 14 mg is a promising option for managing T2D, offering significant improvements in GC, weight management, cardiovascular risk factors, and patient satisfaction.

## Figures and Tables

**Table 1:** Impact of semaglutide on glycemic and anthropometric parameters and body composition.

Endpoint	Mean Difference	SD	IQR Difference	Median After	Mean After	Median Difference	Test Statistic	*p*-Value
HbA1c (%)	−1.24	1.33	1.44	6.72	6.68	−0.74	7.28 *	<0.05
FPG (mg/dL)	−31.01	41.71	56	123.32	123.32	−26.68	265.50 **	<0.05
Weight (kg)	−3.09	5.84	4.5	88.3	86.1	−2.20	4.14 *	<0.05
BMI (kg/m^2^)	−1.19	1.97	1.78	29.61	29.61	−1.19	4.75 *	<0.05
Body water (kg)	−1.66	6.14	2.4	43.02	43.02	−1.66	523 **	<0.05
Fat mass (%)	−0.9	8.01	2.3	29.3	30.74	−0.70	546.5 **	<0.05
Muscle mass (%)	−2.07	8.74	5.4	59.3	57.92	−2.30	1.85 *	0.06

Legend: HbA1c: glycated hemoglobin; FPG: fasting plasma glucose; BMI: body mass index; SD: standard deviation; IQR: interquartile range. Results are statistically significant with a threshold of *p*-value < 0.05. * paired *t*-test; ** Wilcoxon signed-rank test. The oral dosage of semaglutide was 14 mg/day.

**Table 2:** Impact of semaglutide on cardiovascular risk factors.

Endpoint	Mean Difference	SD	IQR Difference	Median After	Mean After	Median Difference	Test Statistic	*p*-Value
SBP (mm/hg)	−12.74	1.33	16.11	126.11	126.11	−13.89	157 **	<0.05
DBP (mm/hg)	−6.39	41.71	10	75.74	75.74	−4.26	132.5 **	<0.05
TC (mg/dL)	−22.19	5.84	37	159.87	159.87	−18.13	3.75 *	<0.05
LDL (mg/dL)	−18.00	1.97	34.5	89	95.5	−24.5	333.5 **	<0.05
HDL (mg/dL)	0.77	6.14	11	44	44.3	−1.07	750.5 **	0.31
TG (mg/dL)	−40.13	8.01	92.32	139	152.55	−27	549 **	<0.05

Legend: SBP: systolic blood pressure; DBP: diastolic blood pressure; TC: total cholesterol; HDL: high-density lipoprotein cholesterol; LDL: low-density lipoprotein cholesterol; TG: triglycerides; SD: standard deviation; IQR: interquartile range; * statistical test: paired *t*-test; ** Wilcoxon signed-rank test. Results are statistically significant with a threshold of *p*-value < 0.05. The oral dosage of semaglutide was 14 mg/day.

**Table 3:** Impact of semaglutide on QoL and treatment satisfaction.

Endpoint	Mean Difference	SD	IQR Difference	Median Difference	Median After	Mean After	Test Statistic	*p*-Value
SF-36 (score)	1.16	1.33	8.18	0	103	101.18	705.5 **	0.17
DTSQ (score)	4.31	41.71	10	4.31	31.02	31.02	445 **	<0.05

Legend: DTSQ: Diabetes Treatment Satisfaction Questionnaire; SF-36: 36-item short-form health survey; SD: standard deviation; CI IQR: interquartile range; ** Wilcoxon signed-rank test. Results statistically significant, with a threshold of *p*-value < 0.05.

## Data Availability

Data supporting this research are available in the Supplementary Materials.

## Supplementary Materials

The following supporting information can be downloaded at https://www.mdpi.com/article/10.3390/jcm13164752/s1, complete data analysis.
