# Supplementary material for: Oral Semaglutide in Type 2 Diabetes: Clinical–Metabolic Outcomes and Quality of Life in Real-World Practice
Source: J Clin Med. 2024 Aug 13;13(16):4752. doi: 10.3390/jcm13164752 (PMC11355440; doi:10.3390/jcm13164752)
Supplement: Supplementary file 1 [file jcm-13-04752-s001.zip › jcm-3158369-supplementary.pdf]

Statistical Analysis Results

Endpoint	Test Type	Mean Difference	Std Deviation	S.E. Mean	95% CI Lower	95% CI Upper	Test Statistic	p-value	Effect Size	Median Difference	IQR Difference	Mean Before	Median Before	IQR Before	Mean After	Median After	IQR After
HbA1c	Paired t-test	-1.24	1.33	0.17	-1.59	-0.9	7.28	0.0	-0.93	-0.74	1.44	7.92	7.46	1.9	6.68	6.72	1.28
Weight	Paired t-test	-3.09	5.84	0.75	-4.59	-1.6	4.14	0.0	-0.53	-2.2	4.5	89.19	90.5	20.1	86.1	88.3	20.7
Body Water	Wilcoxon signed-rank test	-1.66	6.14	0.79	nan	nan	523.0	0.0	0.45	-1.66	2.4	44.68	44.68	11.4	43.02	43.02	12.0
Fat Mass %	Wilcoxon signed-rank test	-0.07	8.01	1.03	nan	nan	546.5	0.01	0.42	-0.7	2.3	30.81	30.0	5.5	30.74	29.3	6.7
Muscle Mass	Paired t-test	-2.07	8.74	1.12	-4.31	0.17	1.85	0.07	-0.24	-2.3	5.4	59.99	61.6	15.5	57.92	59.3	13.7
Quality of Life (SF-36)	Wilcoxon signed-rank test	1.16	14.79	1.89	nan	nan	705.5	0.17	0.25	0.0	8.18	100.02	103.0	9.0	101.18	103.0	9.0
Quality of Life (QoL)	Wilcoxon signed-rank test	4.31	8.75	1.12	nan	nan	445.0	0.0	0.53	4.31	10.0	26.7	26.7	10.0	31.02	31.02	6.0
FPG	Wilcoxon signed-rank test	-31.01	41.71	5.34	nan	nan	265.5	0.0	0.72	-26.68	56.0	154.33	150.0	41.0	123.32	123.32	44.0
BMI	Paired t-test	-1.19	1.96	0.25	-1.7	-0.69	4.75	0.0	-0.61	-1.19	1.78	30.81	30.81	6.04	29.61	29.61	5.32

Systolic BP	Wilcoxon signed-rank test	-12.74	16.53	2.12	nan	nan	157.0	0.0	0.83	-13.89	16.11	138.85	140.0	20.0	126.11	126.11	10.0
Diastolic BP	Wilcoxon signed-rank test	-6.39	12.04	1.54	nan	nan	132.5	0.0	0.86	-4.26	10.0	82.14	80.0	15.0	75.74	75.74	10.0
Total Cholesterol	Paired t-test	-22.19	46.26	5.92	-34.04	-10.34	3.75	0.0	-0.48	-18.13	37.0	182.05	178.0	56.0	159.87	159.87	46.0
LDL Cholesterol	Wilcoxon signed-rank test	-18.0	34.51	4.42	nan	nan	333.5	0.0	0.65	-24.5	34.5	113.5	113.5	44.0	95.5	89.0	47.0
HDL Cholesterol	Wilcoxon signed-rank test	-0.77	9.11	1.17	nan	nan	750.5	0.31	0.21	-1.07	11.0	45.07	45.07	12.0	44.3	44.0	15.0
Triglycerides	Wilcoxon signed-rank test	-40.13	115.11	14.74	nan	nan	549.0	0.01	0.42	-27.0	92.32	192.68	166.0	88.0	152.55	139.0	79.0

Columns Description:

- Endpoint: The specific parameter or health outcome being measured.
- Test Type: The statistical test used to compare the before and after measurements, either a paired t-test or a Wilcoxon signed-rank test.
- Mean Difference: The mean difference between the measurements before and after the intervention.
- Std Deviation: The standard deviation of the differences between the before and after measurements.
- S.E. Mean: The standard error of the mean difference.
- 95% CI Lower: The lower bound of the 95% confidence interval for the mean difference (only applicable for parametric tests).
- 95% CI Upper: The upper bound of the 95% confidence interval for the mean difference (only applicable for parametric tests).
- Test Statistic: The test statistic value for the statistical test performed.
- p-value: The p-value indicating the statistical significance of the test results.
- Effect Size: A measure of the magnitude of the intervention's effect.
- Median Difference: The median difference between the before and after measurements.
- IQR Difference: The interquartile range (IQR) of the differences between the before and after measurements.
- Mean Before: The mean value of the measurements before the intervention.
- Median Before: The median value of the measurements before the intervention.
- IQR Before: The interquartile range (IQR) of the measurements before the intervention.
- Mean After: The mean value of the measurements after the intervention.
- Median After: The median value of the measurements after the intervention.
- IQR After: The interquartile range (IQR) of the measurements after the intervention.

Endpoints Included in the Table:

- HbA1c: Glycated hemoglobin percentage, a measure of blood sugar control.
- Weight: The body weight of the subjects.
- Body Water: The percentage of body water.
- Fat Mass %: The percentage of body fat.
- Muscle Mass: The amount of muscle mass.
- Quality of Life (SF-36): A measure of general health-related quality of life.
- Quality of Life (QoL): A specific quality of life measure.
- FPG: Fasting plasma glucose, a measure of blood sugar levels.
- BMI: Body Mass Index, a measure of body fat based on height and weight.
- Systolic BP: Systolic blood pressure.
- Diastolic BP: Diastolic blood pressure.
- Total Cholesterol: The total cholesterol level in the blood.
- LDL Cholesterol: Low-density lipoprotein cholesterol, often called "bad" cholesterol.
- HDL Cholesterol: High-density lipoprotein cholesterol, often called "good" cholesterol.
- Triglycerides: The level of triglycerides in the blood.
